# Joint Power and Subchannel Allocation for Distributed Storage in Cellular-D2D Underlays

**DOI:** 10.3390/s21238059

**Published:** 2021-12-02

**Authors:** Fengxia Han, Hao Deng, Jianfeng Shi, Hao Jiang

**Affiliations:** 1 School of Software Engineering, Tongji University, Shanghai 201804, China; fengxiahan@tongji.edu.cn; 2 School of Electronic and Information Engineering, Nanjing University of Information Science and Technology, Nanjing 210044, China; jianfeng.shi@nuist.edu.cn; 3 National Mobile Communications Research Laboratory, Southeast University, Nanjing 210096, China; jianghao@nuist.edu.cn; 4 College of Artifcial Intelligence, Nanjing University of Information Science and Technology, Nanjing 210044, China

**Keywords:** wireless distributed storage, cellular-D2D underlay, non-orthogonal multiple access (NOMA), joint resource allocation

## Abstract

Wireless distributed storage is beneficial in the provision of reliable content storage and offloading of cellular traffic. In this paper, we consider a cellular device-to-device (D2D) underlay-based wireless distributed storage system, in which the minimum storage regenerating (MSR) coding combined with the partial downloading scheme is employed. To alleviate burdens on insufficient cellular resources and improve spectral efficiency in densely deployed networks, multiple storage devices can simultaneously use the same uplink cellular subchannel under the non-orthogonal multiple access (NOMA) protocol. Our objective is to minimize the total transmission power for content reconstruction, while guaranteeing the signal-to-interference-plus-noise ratio (SINR) constraints for cellular users by jointly optimizing power and subchannel allocation. To tackle the non-convex combinational program, we decouple the original problem into two subproblems and propose two low-complexity algorithms to efficiently solve them, followed by a joint optimization, implemented by alternately updating the solutions to each subproblem. The numerical results illustrate that our proposed algorithms are capable of performing an exhaustive search with lower computation complexity, and the NOMA-enhanced scheme provides more transmission opportunities for neighbor storage devices, thus significantly reducing the total power consumption.

## 1. Introduction

The explosively growing mobile data traffic has dramatically burdened current wireless networks and posed a great challenge to the future 6G communications. To alleviate the limited wireless bottlenecks, distributed storage over wireless links has been introduced as a promising technique for offloading the ever-increasing cellular traffic [1,2,3]. For a distributed storage system, the popular content files can be pre-stored across multiple distributed storage devices (called content helpers). Users requiring the stored content (content requesters) can directly download them from neighboring content helpers (CHs) instead of from the serving BS, resulting in lower power consumption and content delivery delay [4,5,6].

In practical communication scenarios, storage devices may be individually unreliable when some storage device fails or leaves the network, and thus loses its stored content. To maintain high reliability, there has been a large body of related work [7,8,9,10] researching storage coding schemes to facilitate a reconstruction of the original content, as well as repairing the lost data. Among them, the minimum storage regenerating (MSR) codes invoked in [7] could achieve the optimal tradeoff between repair bandwidth and storage efficiency. However, in most applications of MSR codes, the content requesters (CRs) tend to download all the data stored in specific CHs to reconstruct its desired content [11,12,13]. Considering the limited bandwidth of wireless links between CHs and CRs, it could be more advantageous to allow CRs to download only a small part of the stored symbols from any CH. As proved in [14], a partial downloading scheme could provide more freedom in terms of downloading choices, and consequently consume less power for content reconstruction. Inspired by these ideas, the MSR coding scheme, combined with the partial downloading scheme, will be employed in our proposed wireless distributed storage system.

For more efficient content delivery without additional infrastructure costs, device-to-device (D2D) communications have emerged as a potential candidate for direct transmission between CHs and CRs. For example, our previous work [15] investigated a D2D-assisted wireless distributed storage system to provide power-efficient content delivery while meeting the reliability requirements. Work [16] addressed the repair problem when a D2D device storing data failed and derived the analytical expression for power consumption of data repair, which was verified to be significantly lower compared with the traditional, cellular-only communications. On the other hand, to avoid the incompatibility issues with unlicensed spectrum, reusing the licensed spectrum (i.e., cellular resources) for D2D transmission provides much better spectral efficiency through careful interference coordination. Towards this end, leveraging the cellular-D2D underlay mode for distributed storage systems has attracted increasing interest in the recent literature [17,18,19,20]. Based on graph theory, the optimization of spectrum resource allocation among CHs and cellular users (CUs) has been analyzed in [17,18], targeting the minimization of content reconstruction costs. Considering the mobility and different interests of CHs, the authors in [19,20] focused on socially enabled D2D communications over cellular links. To search for and assign qualified D2D links for content reconstruction, they evaluated the success rate for content delivery based on the statistic social interaction information, as well as the D2D transmission effects on cellular communications. Unfortunately, all the aforementioned work adopted the full downloading scheme to reconstruct the desired content and assumed that different D2D links are orthogonal with each other for mathematical tractability, in which the disadvantages stem from the scarcity of spectrum resources, limiting the number of feasible CHs, and thus may not be applicable in cellular-D2D underlays with densely deployed storage devices.

Differing from the orthogonal multiple access (OMA) technique, non-orthogonal multiple access (NOMA) is able to address both the massive connectivity and spectral efficiency enhancement issue by allowing multiple users to share the same resources simultaneously. Recently, several approaches have been proposed to apply the NOMA technique in D2D-enabled cellular networks for an enhanced system performance. For instance, the work [21,22] considered the non-orthogonal resource-sharing between cellular users and D2D pairs, for which the fractional frequency reuse technique and a cell sectorization method were proposed to mitigate the uplink interference, and both the overall throughput and spectral efficiency were demonstrated to be greatly improved. By delicately designing algorithms for resource allocation under the NOMA protocol, the system sum-rate achieved in [23,24,25,26] greatly outperformed the conventional OMA scheme. The potential benefits of NOMA technology motivated us to reconsider the pattern of spectrum utilization in wireless distributed storage systems, especially when we employ the partial downloading scheme and the available cellular resources are not affordable when solely occupied by each CH. However, to the best of the authors’ knowledge, none of the existing work has been devoted to problems regarding NOMA-enhanced distributed storage in cellular-D2D underlays.

Against this background, we will consider the setting of a wireless distributed storage system in cellular-D2D underlays, where multiple storage devices are allowed to simultaneously reuse the same uplink cellular resources in this paper. To mitigate the uplink interference, a joint optimization on power and subchannel allocation is formulated to minimize the total transmission power while guaranteeing both the SINR constraints at CUs and successful content reconstruction at the CR. Specifically, the original combinational optimization is proposed to be solved by taking the alternative minimization approach, for which a low-complexity greedy-heuristic algorithm and a matching-based algorithm are employed to efficiently deal with each subproblem. In summary, the contributions in this paper are as follows:

- A practical framework for distributed storage in cellular-D2D underlays with the NOMA protocol is proposed, where the MSR coding and partial downloading scheme are combined for more power-efficient choices. The joint optimization of power and subchannel allocation is formulated, which aims to minimize the total transmission power for content reconstruction while guaranteeing the SINR requirements for CUs.
- Given fixed subchannel allocation, a low-complexity power allocation algorithm modified from the greedy-heuristic approach is developed. In particular, a new sorting coefficient is introduced, which considers the interference effects from the CHs to CUs. The simulation results show that our proposed algorithm will closely approach the performance of the exhaustive method, and the newly introduced coefficient will bring a higher transmission rate from CHs rather than from the serving BS, which contributes to the lower power consumption.
- Based on the fixed power allocation, the matching game with externalities is applied to model resource pairing between CHs and CUs, and a low-complexity subchannel allocation algorithm is proposed. Then, the joint optimization can be performed by alternatively updating power and subchannel allocation. Simulation results verify the convergence and near-optimal property of the proposed algorithm, and demonstrate that the NOMA-enhanced transmission scheme and partial downloading can significantly improve the performance gain over the conventional OMA and full downloading scheme.

The remainder of this paper is organized as follows. Section 2 presents the system model for distributed storage in cellular-D2D underlay, and formulates the problem of joint power and subchannel allocation. In Section 3 and Section 4, the original problem is decoupled into two subproblems and then solved. Simulation results are reported in Section 5 to evaluate the performance of our proposed algorithms and investigate the superiority of the NOMA technique as well as the partial downloading scheme. Finally, conclusions are given in Section 6.

## 2. System Model and Problem Formulation

### 2.1. System Description

**Figure 1:**
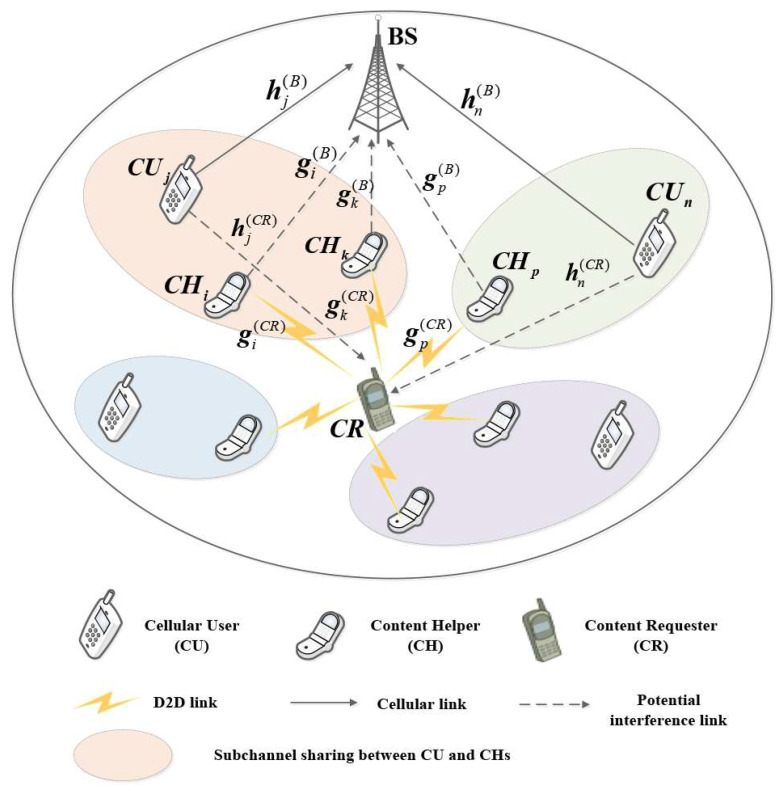
System model for distributed storage in cellular-D2D underlay.

To offload traffic of cellular network and avoid unreliable individual storage devices, we investigated a wireless distributed storage mechanism in cellular-D2D underlays, where a specific content requester (CR) can directly reconstruct its desired content files from multiple adjacent content helpers (CHs) that have pre-stored the content instead of downloading it from the serving BS, as shown in Figure 1. In more detail, we assume that there exist *N* cellular users (CUs), denoted as $\mathrm{CU}=\{CU_{1},\;CU_{2},\;\ldots ,\;CU_{N}\}$, communicating with the BS in traditional cellular links, and assume *M* CHs, denoted as $\mathrm{CH}=\{CH_{1},\;CH_{2},\;\ldots ,\;CH_{M}\}$, attempt to communicate with the CR via D2D links by reusing the uplink cellular subchannels (SCs) occupied by CUs. By further assuming a fully loaded cellular network with available cellular resources denoted by $\mathrm{SC}=\{SC_{1},\;SC_{2},\;\ldots ,\;SC_{N}\}$, each $CU_{j}\in \mathrm{CU}$ for j∈N={1, 2, …, N} is allocated to $SC_{j}\in \mathrm{SC}$ and all SCs are orthogonal. Note that when uplink cellular resources are not sufficient for exclusive assignment, i.e., N≤M, more than one CH may share the same SC to communicate with the CR based on non-orthogonal multiple access (NOMA) protocols.

For i∈M={1, 2, …, M}, let $g_{i}^{\left(CR\right)}$ and $g_{i}^{\left(B\right)}$ denote the signal channel gain from $CH_{i}$ to CR and the interference gain from $CH_{i}$ to the BS, respectively. Similarly, let $h_{j}^{\left(B\right)}$ and $h_{j}^{\left(CR\right)}$ denote the signal channel gain from $CU_{j}$ to the BS and the interference gain from $CU_{j}$ to CR, respectively. In addition, let $\beta_{i,j}$ represent the resource reuse indicator for $CH_{i}\in \mathrm{CH}$ and $SC_{j}\in \mathrm{SC}$, where $\beta_{i,j}=1$ when $CH_{i}$ reuses the resource of $CU_{j}$; otherwise, $\beta_{i,j}=0$. It is assumed that the perfect CSI is available at the serving BS and the considered CR. Then, the received signal-to-interference-plus-noise ratio (SINR) at the BS corresponding to $CU_{j}$ can be expressed as

(1) $$\begin{array}{c} \Gamma_{j}=\frac{Q_{j}{\left|h_{j}^{\left(B\right)}\right|}^{2}}{\sum_{i=1}^{M}\beta_{i,j}P_{i}{\left|g_{i}^{\left(B\right)}\right|}^{2}+\sigma^{2}},\;\;\forall j\in N, \end{array}$$

where $\sigma^{2}$ is the noise variance and $\sum_{i=1}^{M}\beta_{i,j}P_{i}{\left|g_{i}^{\left(B\right)}\right|}^{2}$ is the interference from the CHs sharing the subchannel $SC_{j}$ with $CU_{j}$. Let $Q_{j}$ and $P_{i}$ be the transmission power of $CU_{j}$ and $CH_{i}$, respectively. In this paper, we assume that all involved CUs have fixed transmission power and our objective is to minimize the total transmission power of CHs, i.e., $\sum_{i=1}^{M}P_{i}$, while guaranteeing successful content reconstruction at the CR as well as acceptable communication rates for CUs.

Inspired by the principle of NOMA, for the case in which multiple CHs tend to reuse the same SC to transmit content simultaneously, the technique of successive interference cancellation (SIC) could be employed at the CR to mitigate the inter-user interference. Based on SIC, the messages from stronger communication links will be successively decoded while the other messages from co-channel interferers are all treated as noise. Without a loss of generality, we assume that CUs are geographically closer to the BS and generate less interference to the CR than CHs. Let $M_{j}=\left\{\forall i\in M|\beta_{i,j}=1\right\}$ denote the index set of CHs using $SC_{j}$ with size $t_{j}=\left|M_{j}\right|$, and denote $\pi_{j}\left(t\right)$ with $t\in T_{j}=\left\{1,\;2,\;\ldots ,\;t_{j}\right\}$ as a sort function, indicating the decreasing order of channel coefficients in $M_{j}$, i.e., $|g_{\pi_{j}\left(1\right)}^{\left(CR\right)}{|}^{2}\geq |g_{\pi_{j}\left(2\right)}^{\left(CR\right)}{|}^{2}\geq \cdots \geq {\left|g_{\pi_{j}\left(t_{j}\right)}^{\left(CR\right)}\right|}^{2}$. Then, the received SINR at the CR from each CH can be obtained, following

(2) $$\begin{array}{cc} \; & \gamma_{\pi_{j}\left(1\right)}=\frac{P_{\pi_{j}\left(1\right)}{\left|g_{\pi_{j}\left(1\right)}^{\left(CR\right)}\right|}^{2}}{\sum_{t=2}^{t_{j}}P_{\pi_{j}\left(t\right)}|g_{\pi_{j}\left(t\right)}^{\left(CR\right)}{|}^{2}+Q_{j}{\left|h_{j}^{\left(CR\right)}\right|}^{2}+\sigma^{2}},\\ \; & \gamma_{\pi_{j}\left(2\right)}=\frac{P_{\pi_{j}\left(2\right)}{\left|g_{\pi_{j}\left(2\right)}^{\left(CR\right)}\right|}^{2}}{\sum_{t=3}^{t_{j}}P_{\pi_{j}\left(t\right)}|g_{\pi_{j}\left(t\right)}^{\left(CR\right)}{|}^{2}+Q_{j}{\left|h_{j}^{\left(CR\right)}\right|}^{2}+\sigma^{2}},\\ \; & \vdots \\ \; & \gamma_{\pi_{j}\left(t_{j}\right)}=\frac{P_{\pi_{j}\left(t_{j}\right)}{\left|g_{\pi_{j}\left(t_{j}\right)}^{\left(CR\right)}\right|}^{2}}{Q_{j}{\left|h_{j}^{\left(CR\right)}\right|}^{2}+\sigma^{2}}, \end{array}$$

where $Q_{j}{\left|h_{j}^{\left(CR\right)}\right|}^{2}$ is the interference from the $CU_{j}$ occupying the subchannel resource $SC_{j}$.

By further assuming the minimum transmission unit from each CH to the CR is a symbol containing *B* bits, and setting each subchannel with bandwidth *W* and duration *T*, the number of symbols that can be downloaded from $CH_{\pi_{j}\left(t\right)}$ is equivalent to

(3) $$\begin{array}{c} \mu_{\pi_{j}\left(t\right)}=\frac{WT}{B}\log_{2}\left(1+\gamma_{\pi_{j}\left(t\right)}\right). \end{array}$$

### 2.2. Partial Downloading Scheme

For reliability and efficiency, the desired content of CR is supposed to be encoded and stored in CHs using the minimum storage regenerating (MSR) coding scheme in this paper. Note that, for analytical simplicity, we only focus on the storage and downloading process for each specific content and assume the requested content can always be found in CHs; the content popularity distribution is beyond our scope.

By referring to the MSR coding scheme invoked in [7], the desired content file consisted of *L* symbols, denoted as $s={\left[s^{1},\;s^{2},\;\ldots \;s^{L}\right]}^{T}$, will be stored across *M* distributed CHs. Each $CH_{i}$ for i∈M stores α symbols, denoted as $c_{i}={\left[c_{i}^{1},\;c_{i}^{2},\;\ldots \;c_{i}^{\alpha }\right]}^{T}=E_{i}^{T}s$, where $E_{i}$ is an L×α encoding matrix for $CH_{i}$. If the desired content has already been encoded and stored in *M* CHs following the standard MSR procedure, according to the conventional full downloading scheme [12], the CR can reconstruct the content by downloading all α stored symbols from *K*(K≤M) CHs with

(4) $$\begin{array}{c} K=\lceil L/\alpha \rceil . \end{array}$$

However, due to the channel fading and bandwidth constraints of wireless links in practical scenarios, the CR may not be able to download all the stored symbols from each CH. Considering the exponential nature of the transmission power as a function of the number of downloaded symbols, we propose using the power-efficient partial downloading scheme invented in [14] for distributed storage in cellular-D2D underlays, for which the CR can download only a small portion of the stored symbols from more than *K* CHs when the following condition is satisfied:

(5) $$\begin{array}{c} \sum_{j\in N}\sum_{i\in M}\mu_{i,j}\geq L, \end{array}$$

where $\mu_{i,j}$ is the number of symbols to be downloaded from $CH_{i}$ over $SC_{j}$. After determining the number of downloading symbols $\mu_{i,j}$, the CR can further decide which specific symbols t download by using the symbol selection scheme proposed in [14]. Simulation results in Section 5 will verify the superiority of the partial downloading scheme over the conventional full downloading scheme in reducing power consumption.

### 2.3. Problem Formulation

In this paper, we will investigate the joint optimization of power and subchannel assignment for distributed storage devices in cellular-D2D underlays, which aims to minimize the total transmission power for content reconstruction at the CR while guaranteeing the SINR constraints at CUs. For the acquisition of CSI information, suppose each CU and CH will first send some pilots to the BS and the CR for channel estimation before downloading the desired content. Then, after estimating the channel gains, the CR and the BS will perform the joint optimization and coordinate with each other to ensure the SINR constraints. Finally, the corresponding solutions will be fed back to the CHs to determine the transmission power and subchannel.

Specifically, let $\mu_{\pi_{j}\left(t\right)}$ denote the number of symbols to be downloaded from $CH_{\pi_{j}\left(t\right)}$ over $SC_{j}$. Then, from (2) and (3), we can obtain the following expressions:

(6) $$\begin{array}{cc} \; & \kappa \gamma_{\pi_{j}\left(t_{j}\right)}=\log_{2}\left(1+\frac{P_{\pi_{j}\left(t_{j}\right)}{\left|g_{\pi_{j}\left(t_{j}\right)}^{\left(CR\right)}\right|}^{2}}{Q_{j}{\left|h_{j}^{\left(CR\right)}\right|}^{2}+\sigma^{2}}\right),\\ \; & \kappa \gamma_{\pi_{j}\left(t_{j}-1\right)}=\log_{2}\left(1+\frac{P_{\pi_{j}\left(t_{j}-1\right)}{\left|g_{\pi_{j}\left(t_{j}-1\right)}^{\left(CR\right)}\right|}^{2}}{P_{\pi_{j}\left(t_{j}\right)}|g_{\pi_{j}\left(t_{j}\right)}^{\left(CR\right)}{|}^{2}+Q_{j}{\left|h_{j}^{\left(CR\right)}\right|}^{2}+\sigma^{2}}\right),\\ \; & \vdots \\ \; & \kappa \mu_{\pi_{j}\left(1\right)}=\log_{2}\left(1+\frac{P_{\pi_{j}\left(1\right)}{\left|g_{\pi_{j}\left(1\right)}^{\left(CR\right)}\right|}^{2}}{\sum_{t=2}^{t_{j}}P_{\pi_{j}\left(t\right)}|g_{\pi_{j}\left(t\right)}^{\left(CR\right)}{|}^{2}+Q_{j}{\left|h_{j}^{\left(CR\right)}\right|}^{2}+\sigma^{2}}\right), \end{array}$$

where $\kappa =\frac{B}{WT}$. After solving the requested transmission power $P_{\pi_{j}\left(t_{j}\right)},\;P_{\pi_{j}\left(t_{j}-1\right)},\;\ldots ,\;P_{\pi_{j}\left(1\right)}$ from (6) one by one, the transmission power for transmitting $\mu_{\pi_{j}\left(t\right)}$ symbols from $CH_{\pi_{j}\left(t\right)}$ can generally be expressed as

(7) $$\begin{array}{c} P_{\pi_{j}\left(t\right)}=\frac{(Q_{j}|h_{j}^{\left(CR\right)}{|}^{2}+\sigma^{2})\cdot \left(2^{\kappa \mu_{\pi_{j}\left(t\right)}}-1\right)\cdot 2^{\kappa \sum_{t^{\prime }=t+1}^{t_{j}}\mu_{\pi_{j}\left(t^{\prime }\right)}}}{|g_{\pi_{j}\left(t\right)}^{\left(CR\right)}{|}^{2}}. \end{array}$$

Then, the total transmission power over $SC_{j}$ is given by

(8) $$\begin{array}{c} P_{j}=\sum_{t=1}^{t_{j}}P_{\pi_{j}\left(t\right)},\;\;\forall j\in N. \end{array}$$

Note that the power allocation for CHs should also be delicately designed, without causing severe interference to CUs, which means that the content downloading should be performed with the minimum SINR requirements of CUs guaranteed. Therefore, the joint resource allocation problem can be formulated as follows:

(9) $$\begin{array}{cc} \min_{{\left\{\beta_{i,j},\;\mu_{i,j}\right\}}_{i\in M,j\in N}}\;\;\; & \sum_{j=1}^{N}\;\;P_{j} \end{array}$$

(10) $$\begin{array}{cc} s.t.\;\;\;\;\;\;\;\;\;\;\;\; & \frac{Q_{j}{\left|h_{j}^{\left(B\right)}\right|}^{2}}{\sum_{i=1}^{M}\beta_{i,j}P_{i}{\left|g_{i}^{\left(B\right)}\right|}^{2}+\sigma^{2}}\geq \Gamma_{\min},\;\forall j\in N, \end{array}$$

(11) $$\begin{array}{cc} \; & \beta_{i,j}\in \left\{0,1\right\},\;\forall i\in M,\forall j\in N, \end{array}$$

(12) $$\begin{array}{cc} \; & \sum_{j=1}^{N}\beta_{i,j}\leq 1,\sum_{i=1}^{M}\beta_{i,j}\leq q_{\max},\;\forall i\in M,\forall j\in N, \end{array}$$

(13) $$\begin{array}{cc} \; & \mu_{i,j}\in \left\{0,\;1,\;\cdots \alpha \right\},\;\forall i\in M, \end{array}$$

(14) $$\begin{array}{cc} \; & \sum_{j\in N}\sum_{i\in M}\mu_{i,j}=L, \end{array}$$

where $\Gamma_{\min}$ denotes the SINR thresholds for CUs. Constraint (10) restricts the interference received at cellular links from the D2D links. Constraint (11) and (12) dictate that, at most, one SC can be allocated to each CH and, at most, $q_{max}$ CHs can share the same SC. Constraint (13) and (14) guarantee the successful content reconstruction at the CR. The above formulation is an integer program. According to (7), we find that the variables $\beta_{i,j}$ and $\mu_{i,j}$ are coupled with each other in $P_{i}$, which leads to the non-convexity of constraint (10). To this end, to deal with the combinational problem, in the following, we will decouple the original problem into two subproblems and provide the solutions for (1) power allocation among all CHs; (2) subchannel allocation over all the available SCs. After dealing with each subproblem, a joint algorithm can be then implemented, in which the subchannel and power allocation are performed alternatively until an acceptable suboptimal solution is obtained.

## 3. Power Allocation for Content Reconstruction

In this section, supposing the subchannel allocation is settled, we solve the subproblem of power allocation among all CHs, such that the total transmission power for content reconstruction is minimized. Due to the non-linear constraints in problem (9), the decoupled subproblem of power allocation is still intricate. Therefore, we first consider dropping some of the constraints that complicate the power allocation and provide a greedy-heuristic approach, which has been proven to achieve the optimal solutions to the relaxed problem. After modifying some of the selecting steps, we further propose a suboptimal algorithm with lower computational complexity, which is capable of recovering the originally dropped constraints. Simulation results will show that our proposed power allocation algorithm is almost close to the performance of the exhaustive method.

### 3.1. Optimal Power Allocation for the Relaxed Problem

By fixing the channel allocation variables ${\left\{\beta_{i,j}\right\}}_{i\in M,j\in N}$ and dropping the constraints (10) and (13), the relaxed subproblem of power allocation can be reformulated as

(15) $$\begin{array}{cc} \min_{{\left\{\mu_{\pi_{j}\left(t\right)}\right\}}_{t\in T_{j},j\in N}}\;\;\; & \sum_{j=1}^{N}\;\;P_{j} \end{array}$$

(16) $$\begin{array}{cc} s.t.\;\;\;\; & \mu_{\pi_{j}\left(t\right)}\in Z^{+}\cup \left\{0\right\},\;\forall j\in N,\forall t\in T_{j}, \end{array}$$

(17) $$\begin{array}{cc} \; & \sum_{j=1}^{N}\sum_{t=1}^{t_{j}}\mu_{\pi_{j}\left(t\right)}=L. \end{array}$$

Assume that the number of downloaded symbols from CHs that reuse the cellular channel $SC_{j}$ is defined as $\left[\mu_{\pi_{j}\left(1\right)},\;\mu_{\pi_{j}\left(2\right)},\;\ldots ,\;\mu_{\pi_{j}\left(t_{j}\right)}\right]$; the total transmission power over $SC_{j}$ is then given by

(18) $$\begin{array}{c} P_{j}\left([\mu_{\pi_{j}\left(1\right)},\;\mu_{\pi_{j}\left(2\right)},\;...,\;\mu_{\pi_{j}\left(t_{j}\right)}]\right)=\sum_{t=1}^{t_{j}}P_{\pi_{j}\left(t\right)}, \end{array}$$

where the specific value for $P_{\pi_{j}\left(t\right)}$ can be referred to (7). Before presenting the optimal solutions to the relaxed problem (15), we obtain the following theorem about the optimal choice over each $SC_{j}$.

**Theorem** **1.** *The minimized sum power among CHs over each SC is achieved by downloading all the potential symbols from the CH with the strongest D2D link coefficient, i.e.,*

*$P_{j}\left([\mu_{\pi_{j}\left(1\right)},\;\mu_{\pi_{j}\left(2\right)},\;...,\;\mu_{\pi_{j}\left(t_{j}\right)}]\right)\;\geq \;P_{j}\left([\mu_{\pi_{j}\left(1\right)}^{{}^{\prime }},\;0,\;...,\;0,\;0]\right)$, where $\mu_{\pi_{j}\left(1\right)}^{{}^{\prime }}=\sum_{t=1}^{t_{j}}\mu_{\pi_{j}\left(t\right)}$.*

**Proof.**  Based on (18), we have the following comparison

(19) $$\begin{array}{cc} \; & P_{j}\left([\mu_{\pi_{j}\left(1\right)},\;\mu_{\pi_{j}\left(2\right)},\;\ldots ,\;\mu_{\pi_{j}\left(t_{j}\right)}]\right)-P_{j}\left([\mu_{\pi_{j}\left(1\right)}^{{}^{\prime }},\;0,\;\ldots ,\;0,\;0]\right)\\ \; & =(Q_{j}|h_{j}^{\left(CR\right)}{|}^{2}+\sigma^{2})\times \left\{\sum_{t=1}^{t_{j}}\frac{\left(2^{\kappa \mu_{\pi_{j}\left(t\right)}}-1\right)\cdot 2^{\kappa \sum_{t^{\prime }=t+1}^{t_{j}}\mu_{\pi_{j}\left(t^{\prime }\right)}}}{|g_{\pi_{j}\left(t\right)}^{\left(CR\right)}{|}^{2}}-\left(\frac{2^{\kappa \sum_{t=1}^{t_{j}}\mu_{\pi_{j}\left(t\right)}}-1}{|g_{\pi_{j}\left(1\right)}^{\left(CR\right)}{|}^{2}}\right)\right\}\\ \; & \geq \left\{\frac{2^{\kappa \sum_{t=1}^{t_{j}}\mu_{\pi_{j}\left(t\right)}}}{|g_{\pi_{j}\left(1\right)}^{\left(CR\right)}{|}^{2}}-\frac{2^{\kappa \sum_{t=2}^{t_{j}}\mu_{\pi_{j}\left(t\right)}}}{|g_{\pi_{j}\left(1\right)}^{\left(CR\right)}{|}^{2}}+\cdots +\frac{2^{\kappa \mu_{\pi_{j}\left(t_{j}\right)}}}{|g_{\pi_{j}\left(t_{j}\right)}^{\left(CR\right)}{|}^{2}}-\frac{1}{|g_{\pi_{j}\left(t_{j}\right)}^{\left(CR\right)}{|}^{2}}-\frac{2^{\kappa \sum_{t=1}^{t_{j}}\mu_{\pi_{j}\left(t\right)}}}{|g_{\pi_{j}\left(1\right)}^{\left(CR\right)}{|}^{2}}+\frac{1}{|g_{\pi_{j}\left(1\right)}^{\left(CR\right)}{|}^{2}}\right\}\\ \; & \geq \{\frac{1}{|g_{\pi_{j}\left(1\right)}^{\left(CR\right)}{|}^{2}}+\left(\frac{1}{|g_{\pi_{j}\left(2\right)}^{\left(CR\right)}{|}^{2}}-\frac{1}{|g_{\pi_{j}\left(1\right)}^{\left(CR\right)}{|}^{2}}\right)\cdot 2^{\kappa \sum_{t=2}^{t_{j}}\mu_{\pi_{j}\left(t\right)}}+\cdots \\ \; & \;\;\;+\left(\frac{1}{|g_{\pi_{j}\left(t_{j}\right)}^{\left(CR\right)}{|}^{2}}-\frac{1}{|g_{\pi_{j}\left(t_{j}-1\right)}^{\left(CR\right)}{|}^{2}}\right)\cdot 2^{\kappa \mu_{\pi_{j}\left(t_{j}\right)}}-\frac{1}{|g_{\pi_{j}\left(t_{j}\right)}^{\left(CR\right)}{|}^{2}}\}\\ \; & \geq \left\{\frac{1}{|g_{\pi_{j}\left(1\right)}^{\left(CR\right)}{|}^{2}}+\left(\frac{1}{|g_{\pi_{j}\left(2\right)}^{\left(CR\right)}{|}^{2}}-\frac{1}{|g_{\pi_{j}\left(1\right)}^{\left(CR\right)}{|}^{2}}\right)+\cdots +\left(\frac{1}{|g_{\pi_{j}\left(t_{j}\right)}^{\left(CR\right)}{|}^{2}}-\frac{1}{|g_{\pi_{j}\left(t_{j}-1\right)}^{\left(CR\right)}{|}^{2}}\right)-\frac{1}{|g_{\pi_{j}\left(t_{j}\right)}^{\left(CR\right)}{|}^{2}}\right\}\geq 0 \end{array}$$

Hence, we prove Theorem 1. □

According to Theorem 1, the total power across all available SCs can be minimized by simply downloading symbols from the CHs with the strongest D2D link coefficient over each SC. Subsequently, we present the following result regarding the optimal solution to problem (15).

**Theorem** **2.** *The greedy-heuristic approach shown in Algorithm 1 provides an optimal solution to power allocation to the relaxed problem (15).*

**Proof.**  According to Theorem 1, since the symbol allocation $\left[\mu_{\pi_{j}\left(1\right)},\;\mu_{\pi_{j}\left(2\right)},\;\ldots ,\;\mu_{\pi_{j}\left(t_{j}\right)}\right]$ can always be written in the form of $\left[\mu_{\pi_{j}\left(1\right)}^{{}^{\prime }},\;0,\;\ldots ,\;0,\;0\right]$ to minimize the total power over $SC_{j}$, we simplify the notation of transmit power $P_{j}\left([\mu_{\pi_{j}\left(1\right)}^{{}^{\prime }},\;0,\;\ldots ,\;0,\;0]\right)$ as $P_{j}\left(\mu \right)$. Let $▵P_{j}^{\left(\mu \right)}=P_{j}\left(\mu +1\right)-P_{j}\left(\mu \right)$ denote the power increment for $CH_{\pi_{j}\left(1\right)}$ transmitting μ+1 symbols compared to transmitting μ symbols. By assuming the ultimate number of symbols transmitted over $SC_{j}$ is $\mu_{j}$, the total power across all SCs is given by

(20) $$\begin{array}{c} P_{total}=\sum_{j\in N}P_{j}=\sum_{j\in N}\sum_{\mu =0}^{\mu_{j}-1}▵P_{j}^{\left(\mu \right)}, \end{array}$$

where $P_{total}$ is equivalent to the sum of *L* power increment components. Based on the above definitions, the total power can be minimized by finding *L* smallest power increments among all the candidates ${\left\{▵P_{j}^{\left(\mu \right)}\right\}}_{j\in N,0\leq \mu <L}$. In the following, we prove by induction that the greedy-heuristic approach will select the *L* smallest power increments. Specifically, by computing

(21) $$\begin{array}{cc} \; & ▵P_{j}^{\left(\mu +1\right)}-▵P_{j}^{\left(\mu \right)}\\ \; & =\left\{P_{j}^{\left(\mu +2\right)}-P_{j}^{\left(\mu +1\right)}\right\}-\left\{P_{j}^{\left(\mu +1\right)}-P_{j}^{\left(\mu \right)}\right\}\\ \; & =\frac{\left(Q_{j}|h_{j}^{\left(CR\right)}{|}^{2}+\sigma^{2}\right)}{|g_{\pi_{j}\left(1\right)}^{\left(CR\right)}{|}^{2}}\times \left\{\left(2^{\kappa (\mu +2)}-1\right)-2\left(2^{\kappa (\mu +1)}-1\right)+\left(2^{\kappa \mu }-1\right)\right\}\\ \; & =\;\frac{\left(Q_{j}|h_{j}^{\left(CR\right)}{|}^{2}+\sigma^{2}\right)}{|g_{\pi_{j}\left(1\right)}^{\left(CR\right)}{|}^{2}}\cdot 2^{\kappa \mu }\cdot \left(2^{2\kappa }-2^{\kappa +1}+1\right)\\ \; & =\;\frac{\left(Q_{j}|h_{j}^{\left(CR\right)}{|}^{2}+\sigma^{2}\right)}{|g_{\pi_{j}\left(1\right)}^{\left(CR\right)}{|}^{2}}\cdot 2^{\kappa \mu }\cdot {\left(2^{\kappa }-1\right)}^{2}>0, \end{array}$$

we verify that the power increment $▵P_{j}^{\left(\mu \right)}$ is an increasing function of μ, based on which we can obtain the smallest power increment as

(22) $$\begin{array}{c} \min_{j\in N,0\leq \mu \leq L}▵P_{j}^{\left(\mu \right)}=\min_{j\in N}\min_{0\leq \mu \leq L}▵P_{j}^{\left(\mu \right)}=\min_{j\in N}▵P_{j}^{\left(0\right)}. \end{array}$$

Suppose we have finished the selection of $L^{\prime }$$\left(L^{\prime }<L\right)$ smallest power increments and included all of them in a set $P_{L^{\prime }}$. For j∈N, let $\mu_{j}^{c}=\max\;\left\{\mu :▵P_{j}^{\left(\mu \right)}\in P_{L^{\prime }}\right\}$ represent the latest update after $L^{\prime }$ selection. Due to the increasing property of $▵P_{j}^{\left(\mu \right)}$ with μ, we have $▵P_{j}^{\left(\mu \right)}\in P_{L^{\prime }}$ for $\mu \leq \mu_{j}^{c}$. Therefore, the $L^{\prime }+1$ smallest power increment should be illustrated as

(23) $$\begin{array}{c} \min_{j\in N}\min_{\mu >\mu_{j}^{c}}▵P_{j}^{\left(\mu \right)}=\min_{j\in N}▵P_{j}^{\left(\mu_{j}^{c}+1\right)}, \end{array}$$

which is exactly the selection that Algorithm 1 makes. This process will continue to proceed until all the *L* smallest power increment components are observed and, as a consequence, we obtain the optimal solutions to problem (15). □

To illustrate the convergence of Algorithm 1, we observe that the iterations will terminate when $\sum_{j\in N}\mu_{\pi_{j}\left(1\right)}=L$. Since, for each greedy-heuristic search, the value of $\sum_{j\in N}\mu_{\pi_{j}\left(1\right)}=L$ will always be increased by 1, the termination conditions can be satisfied soon after *L* iterations. Besides, given the fact that the desired content size *L* is usually finite, Algorithm 1 will converge to the optimal solutions after *L* iterations.
**Algorithm 1** Greedy-heuristic approach to optimally solve the relaxed problem (15)1:Initialize $\mu_{\pi_{j}\left(t\right)}=0$ for $t\in T_{j},j\in N$.2:**while** $\sum_{j\in N}\mu_{\pi_{j}\left(1\right)}<L$ **do**3:   **for** j∈N **do**4:      Compute $▵P_{j}=P_{j}\left([\mu_{\pi_{j}\left(1\right)}+1,\;0,\;...,\;0]\right)-P_{j}\left([\mu_{\pi_{j}\left(1\right)},\;0,\;...,\;0]\right)$5:   **end for**6:   Find $j^{*}=\arg\min_{j\in N}▵P_{j}$.7:   Update $\mu_{\pi_{j^{*}}\left(1\right)}=\mu_{\pi_{j^{*}}\left(1\right)}+1$.8:**end while**9:Output $\mu_{\pi_{j}\left(t\right)}$ for $t\in T_{j},j\in N$.

### 3.2. Suboptimal Algorithm for Power Allocation

In the previous subsection, we offer Algorithm 1 to optimize the relaxed power allocation problem (15), which has dropped the original constraints (10) and (13). To proceed with the provision of an algorithm considering both the MSR coding constraints and the SINR constraints for CUs, we need to make some adjustments to Algorithm 1.

#### 3.2.1. Constraints for MSR Coding Scheme

As stated in Section 2.2, by employing the MSR coding scheme for distributed storage, each CH only stores α symbols obtained from a linear combination of desired content, thus leading to the constraint for the maximum number of symbols downloaded from each CH, i.e., $\mu_{i,j}\leq \alpha$ for i∈M and j∈N. However, the outputs of Algorithm 1 tend to violate such constraints due to the assumption that only the CH with the strongest link coefficient would transmit symbols. Therefore, at each iteration, we need to check whether the updated allocation satisfies the MSR constraint. To be specific, let $U_{j}={\left\{\mu_{i,j}\right\}}_{i\in M_{j}}$ denote the current allocation over $SC_{j}$; the eligible set under the MSR constraint should conform to

(24) $$\begin{array}{c} C_{MSR}=\left\{U_{j}|\mu_{i,j}\in Z^{+}\cup \left\{0\right\},\mu_{i,j}\leq \alpha ,\forall i\in M_{j}\right\} \end{array}$$

#### 3.2.2. Constrains for SINR Requirements

Except for the MSR constraint, before the gradual increase in the number of downloaded symbols, we should also check whether the SINR requirements for all CUs are fulfilled. According to (1), the feasible set of symbol allocations that meets the SINR thresholds can be expressed as

(25) $$\begin{array}{c} C_{SINR}=\{U_{j}|\sum_{i\in M_{j}}P_{i,j}|g_{i}^{\left(B\right)}{|}^{2}\leq \frac{Q_{j}{\left|h_{j}^{\left(B\right)}\right|}^{2}}{\Gamma_{min}}-\sigma^{2}\}. \end{array}$$

In addition, for the case violating the SINR constraint, we make the following remark:

**Remark** **1.** *If the SINR conditions are not met, regardless of how the power allocation is assigned, the desired content of CR will be downloaded from the serving BS.*

Since transmitting symbols from the BS may cause more power consumption as well as waste the available cellular links, to maintain the stability of the cellular-D2D underlay and minimize the total transmission power for content reconstruction, we should try to reduce the interference from CHs to CUs as much as possible.

#### 3.2.3. Low-Complexity Power Allocation Algorithm

In this subsection, we will propose a low-complexity power allocation algorithm which is capable of including both MSR and SINR constraints while reaching near-optimal solutions. To achieve a better trade-off between the power consumption for content reconstruction and the interference effects on CUs caused by CHs, we introduce a new sort function $\omega_{j}\left(\cdot \right)$, which specifies the priority of CH selection over $SC_{j}$ and defines a relative coefficient $\eta_{\omega_{j}\left(t\right)}=|g_{\omega_{j}\left(t\right)}^{\left(CR\right)}{|}^{2}/{\left|g_{\omega_{j}\left(t\right)}^{\left(B\right)}\right|}^{2}$ such that $\eta_{\omega_{j}\left(1\right)}\geq \eta_{\omega_{j}\left(2\right)}\geq \cdots \geq \eta_{\omega_{j}\left(t_{j}\right)}$. Simulations in Section 5 will demonstrate the rationality and superiority for a selection of $\omega_{j}\left(\cdot \right)$ instead of $\pi_{j}\left(\cdot \right)$. We denote $k=[k_{1},\;k_{2},\;\ldots ,\;k_{N}]$ as an index set indicating the current selection order of $CH_{\omega_{j}\left(k_{j}\right)}$ over $SC_{j}$. Given these definitions, our proposed low-complexity power allocation approach is shown in Algorithm 2.
**Algorithm 2** Low-complexity Power Allocation1:Initialize $U_{j}=0$ for j∈N, $k=1_{N}$ and Ind=1.2:**while** $\sum_{j\in N}\sum_{t\in T_{j}}\mu_{\omega_{j}\left(t\right)}<L$ **do**3:     Set $▵P_{j}=Inf$ for j∈N4:     **for** j∈N **do**5:           **if** $\mu_{\omega_{j}\left(k_{j}\right)}=\alpha$ **Then**6:                  $k_{j}=k_{j}+1$7:           **end if**8:           Get $U_{j}^{{}^{\prime }}$ by modifying $\mu_{\omega_{j}\left(k_{j}\right)}\in U_{j}$ to $\mu_{\omega_{j}\left(k_{j}\right)}+1$9:           **if** $U_{j}^{{}^{\prime }}\in C_{SINR}$ and $U_{j}^{{}^{\prime }}\in C_{MSR}$ **then**10:                  Compute $▵P_{j}=P_{j}\left(U_{j}^{{}^{\prime }}\right)-P_{j}\left(U_{j}\right)$11:           **end if**12:     **end for**13:     **if** $\min_{j\in N}▵P_{j}>10^{6}$ **then**14:           Ind←0 and $P_{total}\leftarrow P_{BS}$15:           **break**;16:     **else**17:           Find $j^{*}=\arg\min_{j\in N}▵P_{j}$18:           Update $U_{j^{*}}\leftarrow U_{j^{*}}^{{}^{\prime }}$19:     **end if**20:**end while**21:**if** Ind=1 **then**22:     $P_{total}\leftarrow \sum_{j=1}^{N}\;\;P_{j}$23:**end if**

In Algorithm 2, we extend the idea of Algorithm 1, which gradually increases the number of symbols downloaded from the last selected CH until the total number of symbols meets the demand for reconstructing the desired content, i.e., $\sum_{j\in N}\left|U_{j}\right|=L$. However, different from Algorithm 1, which only takes the minimized power increment as the measure for the optimal choice at each iteration, Algorithm 2 will check whether the potential variation, denoted by $U_{j}^{{}^{\prime }}$, satisfies the constraints of $C_{MSR}$ and $C_{SINR}$. If there no feasible solution exists, i.e., Ind=0, the CR will download its desired content from the serving BS.

Moreover, to guarantee the superior performance in terms of sum power minimization and avoid high computation complexity, as exhibited in an exhaustive search, which attempts to traverse all the feasible CHs in each iteration, we developed an index set k and limited each selection of CH over $SC_{j}$ to $CH_{\omega_{j}\left(k_{j}\right)}$. To this end, we only need to compute one power increment for each $SC_{j}$ and find the smallest power increment among $SC_{j}\in SC$, rather than compute the power increment of all potential CHs. In this case, the following remark is clear:

**Remark** **2.** *By specifying the selection order and limiting the selection set, Algorithm 2 will significantly reduce the computation complexity, especially when M≫N.*

**Proof.**  Note that the total number of iterations for Algorithm 2 is related to the content size *L*, the complexity is mainly dependent on the calculation of power increment. For the conventional greedy search, this calculation will be performed *M* times for all candidate CHs at each iteration. For Algorithm 2, since the CHs are presupposed to be allocated/grouped into *N* SCs, and only the power increments concerning ${\left\{CH_{\omega_{j}\left(k_{j}\right)}\right\}}_{j\in N}$ need to be calculated at each iteration, the proposed Algorithm 2 only induces a linear complexity of O(LN). □

## 4. Subchannel Allocation Based on Matching Theory

In this section, by assuming that the power allocation of each CH has been addressed, we formulate the subproblem of subchannel allocation to minimize the total transmission power as the following:

(26) $$\begin{array}{cc} \min_{{\left\{\beta_{i,j}\right\}}_{i\in M,j\in N}}\;\;\; & \sum_{i=1}^{M}\;\;P_{i} \end{array}$$

(27) $$\begin{array}{cc} s.t.\;\;\;\; & \frac{Q_{j}{\left|h_{j}^{\left(B\right)}\right|}^{2}}{\sum_{i=1}^{M}\beta_{i,j}P_{i}{\left|g_{i}^{\left(B\right)}\right|}^{2}+\sigma^{2}}\geq \Gamma_{\min},\;\forall j\in N, \end{array}$$

(28) $$\begin{array}{cc} \; & \beta_{i,j}\in \left\{0,1\right\},\;\forall i\in M,\forall j\in N, \end{array}$$

(29) $$\begin{array}{cc} \; & \sum_{j=1}^{N}\beta_{i,j}\leq 1,\sum_{i=1}^{M}\beta_{i,j}\leq q_{max},\;\forall i\in M,\forall j\in N. \end{array}$$

Constraint (27) guarantees the SINR requirements for CUs. Constraint (28) shows that the value of $\beta_{i,j}$ should be either 0 or 1. Constraint (29) indicates that each CH can be assigned, at most, one SC, while each SC can be allocated to no more than $q_{max}$ CHs. The above-formulated subproblem is still a combinational problem and the complexity of the exhaustive method will exponentially increase with the number of CHs and SCs. Therefore, we consider employing the many-to-one two-sided matching theory [27] to efficiently solve the above problem. In the following, we will first introduce some definitions and notations for the proposed matching model and then develop a low-complexity algorithm to obtain solutions to the subchannel allocation problem.

### 4.1. Many-to-One Matching Model and Notations

We first define the proposed matching model between two disjointed sets CH and SC. Specifically, if $SC_{j}$ is allocated to $CH_{i}$, we say $SC_{j}$ and $CH_{i}$ are matched with each other and form a matching pair $\left(CH_{i},SC_{j}\right)$. Then, a complete matching is defined as the set of all the matching pairs of SC allocated to CH, and formally presented as the following:

**Definition** **1.** *Given two disjoint sets SC and CH, a many-to-one matching* Ψ *is a function from the set SC∪CH∪∅ into the set of all subsets of SC∪CH∪∅ such that, for every $CH_{i}\in \mathrm{CH}$ and $SC_{j}\in \mathrm{SC}$:*

*1.*  *$\Psi (CH_{i})\subseteq \mathrm{SC}\cup \emptyset$ with size $|\Psi (CH_{i})|\leq 1$;*
*2.*  *$\Psi (SC_{j})\subseteq \mathrm{CH}\cup \emptyset$ with size $|\Psi \left(SC_{j}\right)|\leq q_{max}$;*
*3.*  *$SC_{j}\in \Psi \left(CH_{i}\right)\Leftrightarrow CH_{i}\in \Psi \left(SC_{j}\right)$.*

Based on the Definition 1, our objective is to determine the optimal matching function that minimizes the total transmission power among all CHs, as shown in (26). Thus, the decision process of each optimal matching pair should depend on the resulted power consumption. By observing the relationship between the value of $P_{i}$ and the value of variable $\beta_{i,j}$, we found that the power consumption of each CH is not only dependent on its matched SC, but also related to the set of other CHs sharing the same SC, which leads to the following remark:

**Remark** **3.** *The matching model formulated between CH and CR is a many-to-one matching game with externalities, also known as the peer effects [28].*

Influenced by peer effects, the transmission power $P_{i}$ for i∈M relies on the current choice of matching function or termed as matching status, and the outcome may be changed according to their co-channel peers under different matching statuses. To deal with such peer effects, we introduce the concept of swap matching to adjust the matching status, as shown below:

**Definition** **2.** *Given a matching function Ψ including pairs $\left(CH_{i},SC_{j}\right)$ and $\left(CH_{p},SC_{n}\right)$, a swap matching is defined by the function $\Psi^{{}^{\prime }}=\Psi \;\backslash \;\left\{\left(CH_{i},SC_{j}\right),\left(CH_{p},SC_{n}\right)\right\}\;\cup \;\left\{\left(CH_{i},SC_{n}\right),\left(CH_{p},SC_{j}\right)\right\}$.*

Based on Definition 2, a swap matching $\Psi^{{}^{\prime }}$ is directly generated by exchanging two allocated SCs of Ψ, while keeping all the other matching pairs the same. Note that one of the CHs involved in the swap can be a “hole” (denoted by $CH_{p}=O$), thus allowing for a single $CH_{i}$ matched with $SC_{n}$ when $|\Psi \left(SC_{n}\right)|<q_{max}$ and leaving an open spot for $SC_{j}=\Psi \left(CH_{i}\right)$. Similarly, one of the SCs involved in the swap can also be a “hole” when $\Psi (CH_{i})=\emptyset$, thus allowing for unmatched $CH_{i}$ to be active.

However, not all the swap operations are beneficial compared to the original matching status. To indicate whether a specific swap operation is necessary and approved, we further introduce the concept of swap-blocking pair as follows:

**Definition** **3.** *Given a matching function* Ψ *with $\left(CH_{i},SC_{j}\right)$ and $\left(CH_{p},SC_{n}\right)$, a pair $\left(CH_{i},CH_{p}\right)$ is defined as a swap-blocking pair if, and only if, it satisfies*

*1.*  *$\forall k\in \left\{CH_{i},CH_{p},SC_{j},SC_{n}\right\},U_{k}\left(\Psi^{{}^{\prime }}\right)\geq U_{k}\left(\Psi \right)$;*
*2.*  *$\exists k\in \left\{CH_{i},CH_{p},SC_{j},SC_{n}\right\},U_{k}\left(\Psi^{{}^{\prime }}\right)>U_{k}\left(\Psi \right)$,*

*where $U_{k}\left(\Psi \right)$ represents the utility of CHs or SCs under matching Ψ and, in our paper, has the following definitions:*

*1.*  *For each $CH_{i}\in \mathrm{CH}$, the utility is defined as the negative power consumption of $CH_{i}$ when it occupies $SC_{j}$ with $SC_{j}=\Psi \left(CH_{i}\right)$, which can be expressed as*
  (30) $$\begin{array}{c} U_{CH_{i}}\left(\Psi \right)=-P_{CH_{i}}; \end{array}$$
*2.*  *For each $SC_{j}\in \mathrm{SC}$, the utility is defined as the negative sum power of all the CHs sharing $SC_{j}$, given by*
  (31) $$\begin{array}{c} U_{SC_{j}}\left(\Psi \right)=-P_{SC_{j}}=\sum_{CH_{t}\in \Psi \left(SC_{j}\right)}-P_{CH_{t}}. \end{array}$$

As proved in [29], a two-sided exchange-stable (2ES) matching always exists in the proposed matching model with peer effects. To reach such 2ES matching, swap operations should be kept approved between the swap-blocking pairs until there is no swap-blocking pair. Through multiple swap matchings, the peer effects can be handled and we can obtain a final stable matching status.

### 4.2. Low-Complexity Subchannel Allocation Algorithm

In this subsection, we propose a low-complexity algorithm to efficiently solve the subchannel allocation, as shown in Algorithm 3, which is equivalent to the process of finding a 2ES matching $\Psi^{*}$ between two disjoint sets CH and SC.
**Algorithm 3** Low-complexity Subchannel Allocation1:Initialize a random matching function $\Psi =\Psi^{0}$ between SC and CH, and set flag=1.2:**while** flag=1 **do**3:    Set flag←04:        **for** $\forall SC_{j}\in \mathrm{SC}$ and $\forall SC_{n}\in \mathrm{SC}\backslash SC_{j}$ **do**5:            **for** $\forall CH_{i}\in \Psi \left(SC_{j}\right)\cup O$ and $\forall \;CH_{p}\in \Psi \left(SC_{n}\right)\cup O$ with O representing the open spot **do**6:                **if** $\left(CH_{i},CH_{p}\right)$ is a swap-blocking pair and (27) is satisfied **then**7:                    Perform swap matching between $\left(CH_{i},CH_{p}\right)$ and update $\Psi \leftarrow \Psi^{{}^{\prime }}$.8:                    Change flag←19:                    **goto** step 4)10:                **end if**11:            **end for**12:        **end for**13:**end while**14:Output the 2ES matching $\Psi^{*}$.

The key idea of Algorithm 3 is to keep executing swap operations until there no swap-blocking pair is recorded by the indicator flag. Moreover, to satisfy the SINR constraints in (27), each time before approving the swap matching, we should also check whether the SINR conditions for all CUs are violated. Through Algorithm 3, we can finally obtain a 2ES matching $\Psi^{*}$, which also constitutes the suboptimal solution to the problem (26). Simulation results in Section 5 will show that the subchannel allocation solutions obtained from Algorithm 3 can approach the optimal solutions obtained by an exhaustive search.

Note that the output $\Psi^{*}$ of Algorithm 3 is not guaranteed to be the global optimal matching. For example, given a matching Ψ with $\Psi \left(CH_{i}\right)=SC_{j}$ and $\Psi \left(CH_{p}\right)=SC_{n}$, if a swap matching $\Psi^{{}^{\prime }}$ satisfies $U_{CH_{i}}\left(\Psi^{{}^{\prime }}\right)<U_{CH_{i}}\left(\Psi \right)$, $U_{SC_{j}}\left(\Psi^{{}^{\prime }}\right)>U_{SC_{j}}\left(\Psi \right)$ and $U_{SC_{n}}\left(\Psi^{{}^{\prime }}\right)>U_{SC_{n}}\left(\Psi \right)$, the sum power will be further reduced after swap operation, but this swap matching is not approved in Algorithm 3 according to Definition 3. In this case, forcing the swap operation to happen may lead to a one-sided exchange matching with weaker stability.

To illustrate the convergence of Algorithm 3, we find that there are, at most, $\frac{N(N-1)}{2}q_{\max}^{2}$ swap-blocking pairs to be checked during each iteration, and each swap matching operation will further reduce the sum power. In this way, given the finite number of *N* and since the sum power has a lower bound, the proposed Algorithm 3 will finally converge to a stable matching status after limited iterations. Moreover, suppose the total number of iterations is given by *I*, then the computation complexity of Algorithm 3 is $O(IN\left(N-1\right)q_{\max}^{2})$.

### 4.3. Joint Power and Subchannel Allocation Algorithm

Based on the previously proposed Algorithms 2 and 3, the joint power and subchannel allocation algorithm can be presented as shown in Algorithm 4. In the initialization phase, a random subchannel matching is given. Then, the power allocation and subchannel allocation are performed under the constraint of the maximum number of iterations $l_{\max}$.
**Algorithm 4** Low-complexity Subchannel Allocation1:Initialize a random subchannel matching function $\Psi =\Psi^{\left(0\right)}$ between SC and CH.2:Initialize the iteration index l=1.3:**while** $l<l_{\max}$ **do**4:    Update the power allocation $P_{i}$ and $U_{j}$ for i∈M,j∈N with fixed $\Psi^{\left(l-1\right)}$ using Algorithm 2.5:    Update the subchannel matching function $\Psi^{\left(l\right)}$ under current power status using Algorithm 3.6:    Update l←l+17:**end while**

## 5. Numerical Results

We consider a wireless distributed storage system in cellular-D2D underlays, for which a specific CR intends to download and reconstruct the desired content from M=8 CHs underlaid with N=4 CUs. Each CH has a storage capacity of α=3 and the original content size is set as L=12. Assume that the distance between the CR and any CH is d1=0.5; the distance between the serving BS and any CH is d2=1.5, the distance between the CR and any CU is d3=1; and the distance between BS and any CU is d4=1.2. Then, the channel gain can be modeled as the complex Gaussian random variable $N_{C}\left(0,d^{-2}\right)$. We further assume that the transmit power for each CU is fixed with $Q_{j}=3$ for j∈N, and the minimum SINR threshold for CUs is set as 0.5. In addition, the transmit power from BS is set to be 100, the system coefficient and the noise power are set as κ=1 and $\sigma^{2}=0.5$, respectively.

In this section, we will first evaluate the performance of our proposed algorithms through simulations, i.e., Algorithm 2 for the subproblem of power allocation and Algorithm 3 for the subproblem of channel allocation. Then, by using Algorithm 4 to jointly perform the power and subchannel allocation, we will further investigate the superiority of the partial downloading scheme as well as the NOMA-enhanced transmission scheme in our proposed cellular-D2D underlay.

### 5.1. Property of the Proposed Algorithms

**Figure 2:**
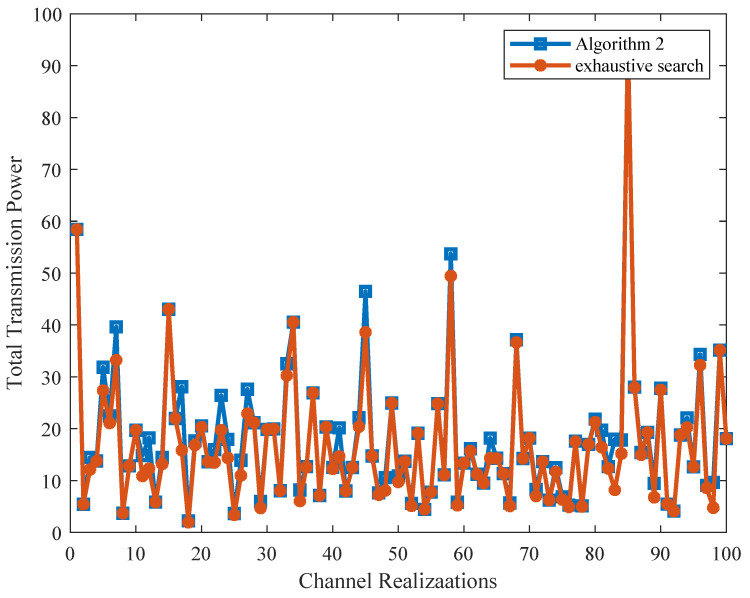
Total transmission power obtained from Algorithm 2 and the exhaustive search.

**Figure 3:**
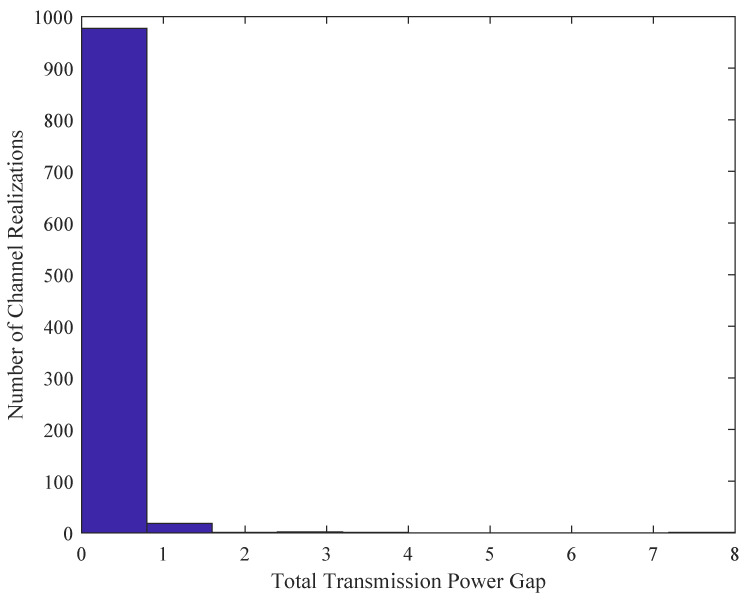
Statistic histogram of the power gap between Algorithm 2 and the exhaustive search.

By randomly fixing a subchannel allocation status, we firstly demonstrate the near-optimal performance of Algorithm 2 for power allocation. Figure 2 plots the total transmission power obtained from our proposed Algorithm 2 for 100 channel realizations. The exhaustive search is also provided as a benchmark for comparison. It can be observed that most of the solutions of Algorithm 2 nearly attain the performance upper bound, i.e., the optimal solutions for power allocation. Figure 3 further shows the statistic histogram of the power gap between Algorithm 2 and the exhaustive search for 1000 channel realizations. As can be observed, around ninety-eight percent of the results obtained from Algorithm 2 are close to the optimal results.

**Figure 4:**
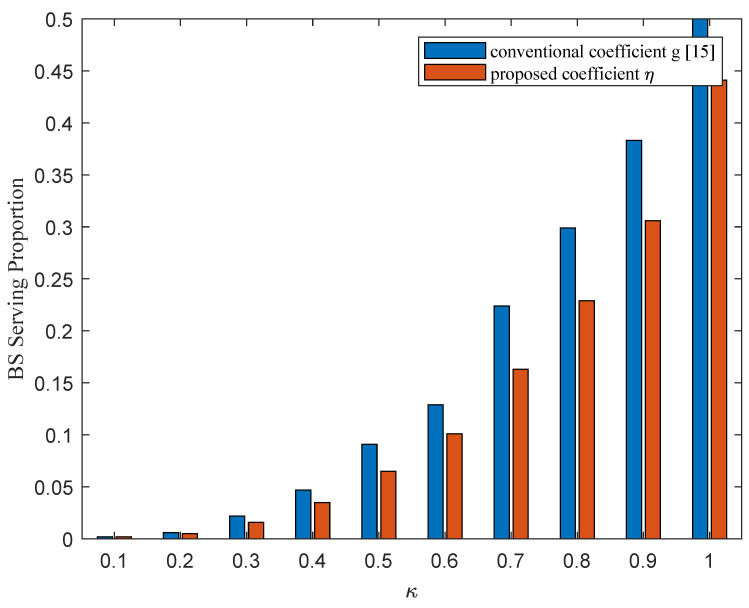
BS serving proportion by using different channel coefficient with SNR = 2 dB.

**Figure 5:**
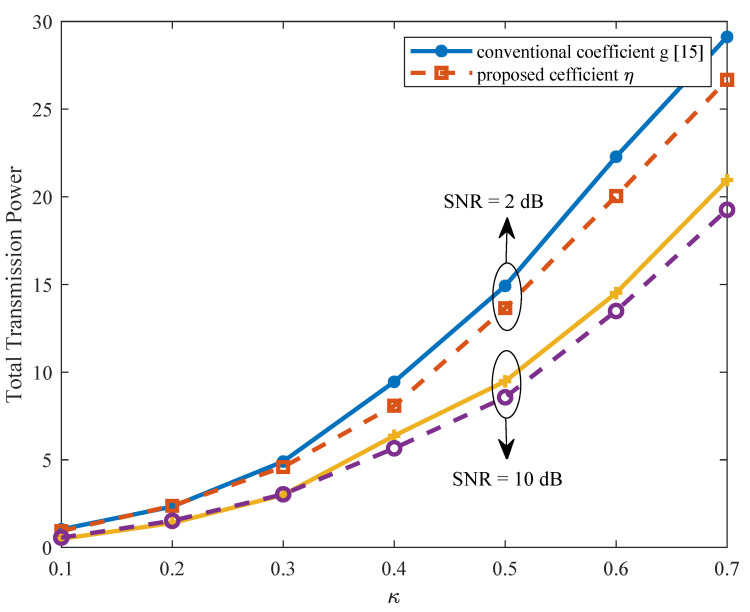
Total transmission power by using different channel coefficient.

Then, we verify the rationality and effectiveness to introduce the newly relative coefficient $\eta_{i}=|g_{i}^{\left(CR\right)}{|}^{2}/{\left|g_{i}^{\left(B\right)}\right|}^{2}$ (denoted by “η”) to specify the selecting order at each iteration in Algorithm 2, instead of using the channel coefficient $|g_{i}^{\left(CR\right)}{|}^{2}$ (“denoted by *g*”), which is usually chosen for the D2D case, as in the work [15]. Figure 4 illustrates the proportion out of all 10,000 desired contents that need to be downloaded from the serving BS due to SINR constraints (denote by “BS Serving Proportion”). From Figure 4, we find out that using the relative coefficient η could guarantee that more content files are downloaded from neighbor CHs rather than from the BS, especially with larger κ, which means that the available bandwidth *W* is limited. Since the BS transmission power is usually much higher, the reduced BS serving proportion would imply a reduced total power consumption for content reconstruction, as can be verified in Figure 5. This makes sense, since the interference effects from CHs to CUs are considered when we select CHs following the order indicated by η, while the original coefficient *g* omits the co-channel interference from CHs to CUs, and thus may violate the SINR constraints.

**Figure 6:**
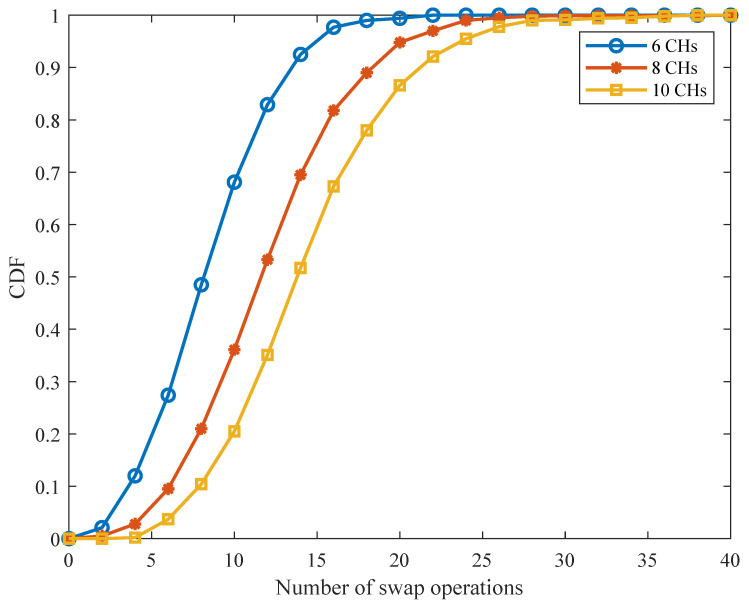
CDF of the number of swap operations in Algorithm 3.

**Figure 7:**
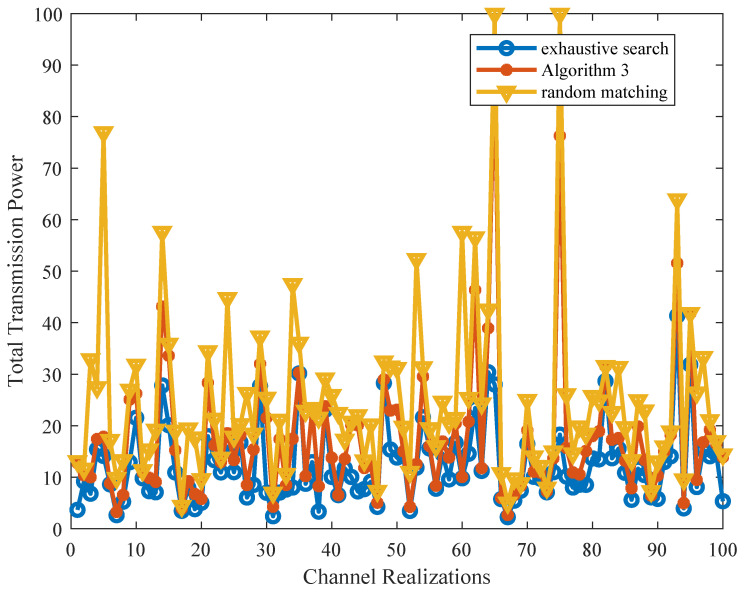
Total transmission power obtained from Algorithm 3 versus the random matching and exhaustive search.

Next, given the fixed power allocation among all CHs, we evaluate the convergence and optimality of Algorithm 3 for subchannel allocation, in which we assume that no more than $q_{max}=3$ CHs are allowed to share the same SC. Figure 6 shows the cumulative distribution function (CDF) of the requested number of swap operations for Algorithm 3 to converge. We observe that Algorithm 3 can always converge within a small number of iterations and the convergence speed will become faster with a decreased number of CHs. Figure 7 plots the total transmission power obtained from Algorithm 3 for 100 channel realizations. The performances of the exhaustive search and the random pairing between CHs and SCs (denoted by “random matching”) are also plotted. It can be seen that the proposed Algorithm 3 brings a greater performance gain over the random matching. Meanwhile, Algorithm 3 is shown to be capable of approximately reaching the optimal results obtained by an exhaustive search in most cases, which, nevertheless, requires lower computational complexity.

### 5.2. Superiority of the Proposed Transmission Schemes

**Figure 8:**
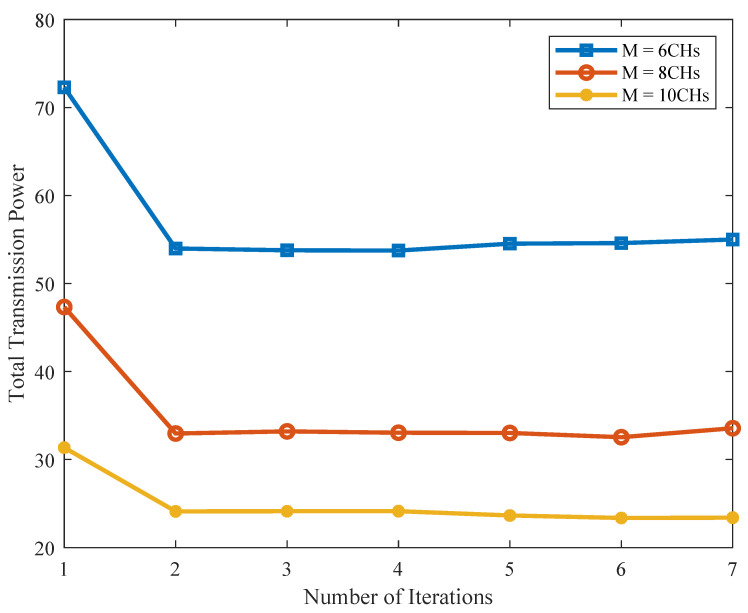
The convergence behaviour of Algorithm 4.

Before demonstrating the superiority of the partial downloading scheme and the NOMA-enhanced transmission scheme for our considered distributed storage systems, we first verify the convergence of the joint power and subchannel allocation optimization, i.e., Algorithm 4, which alternatively implements Algorithms 2 and 3 under the maximum number of iterations $l_{\max}=10$. Figure 8 describes the convergence behavior of Algorithm 4 as the iterative procedure executes, from which we can see that Algorithm 4 will converge after a limited number of iterations.

**Figure 9:**
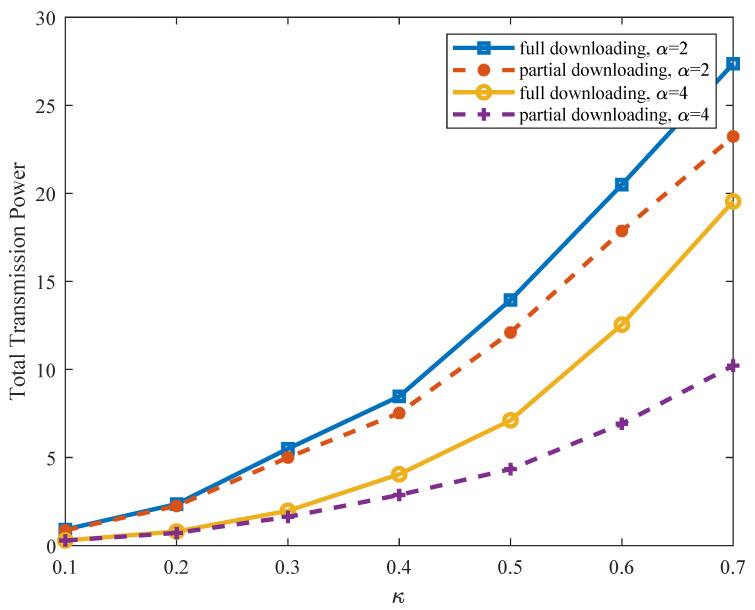
Total transmission power by using the partial downloading versus full downloading.

Then, we exploit the potential benefits of the partial downloading scheme over the conventional full downloading scheme [16]. Figure 9 compares the total transmission power for content reconstruction by using the partial downloading scheme with the full downloading scheme, where the full downloading scheme is achieved by exhaustively searching the optimal L/α CHs and downloading all their stored symbols. As can be observed in Figure 9, the proposed partial downloading scheme can significantly reduce the total transmission power, especially with an increased number of stored symbols α and restricted channel condition κ, in which case the partial downloading scheme provides more freedom of downloading choices and consequently alleviates the exponential increment of transmission power with the number of downloaded symbols.

**Figure 10:**
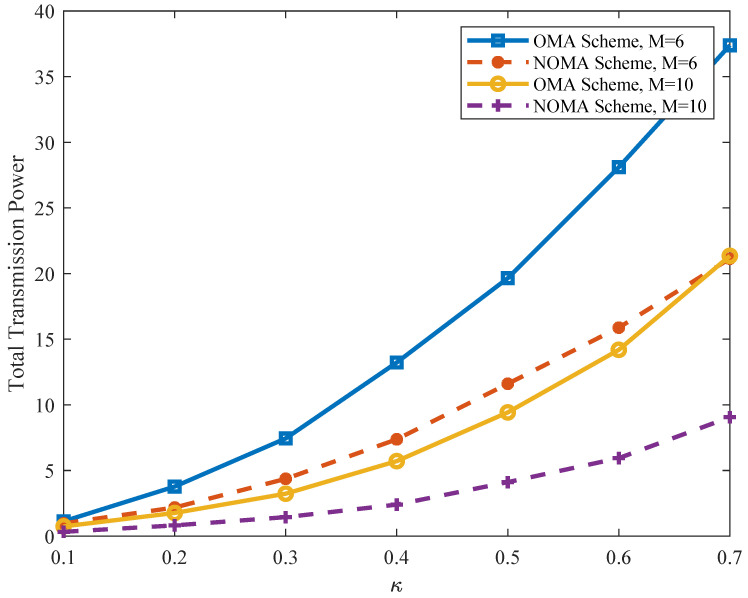
Total transmission power under the NOMA-enhanced scheme versus the OMA scheme.

Finally, Figure 10 shows the total power consumption by using the NOMA-enhanced transmission scheme versus the conventional OMA-based transmission scheme for distributed storage in cellular-D2D underlays. It can be seen that the performance of the NOMA transmission scheme outperforms the OMA scheme with all possible κ values. This is reasonable because, differently from the OMA scheme, for which each SC is only allocated to one CH, applying the NOMA protocol allows subchannel sharing by multiple CHs, and thus improves the resource utilization.

## 6. Conclusions

In this paper, we studied the joint optimization of power and subchannel allocation for wireless distributed storage in cellular-D2D underlays, where the MSR coding and the power-saving partial downloading scheme are employed for content reconstruction. Since the formulated problem was a non-convex combinational optimization, we have decoupled it into two subproblems, i.e., power allocation and subchannel allocation problems. Given a fixed subchannel allocation, a low-complexity, greedy-heuristic algorithm was proposed to solve the power allocation problem. Based on the power allocation results, a matching model with externalities was introduced and a corresponding swap matching algorithm was offered to deal with the subchannel allocation problem. Then, we alternatively performed power and subchannel allocation to obtain the joint optimization. The simulation results verified the convergence as well as the near-optimal property of our proposed algorithms. In addition, it was also shown that the partial-downloading approach outperformed the conventional full-downloading approach, and the NOMA-enhanced distributed storage achieved a larger performance gain.

## Data Availability

Not applicable.
